# The prevalence and outcomes of frail older adults in clinical trials in multiple myeloma: A systematic review

**DOI:** 10.1038/s41408-022-00779-2

**Published:** 2023-01-05

**Authors:** Hira Mian, Arleigh McCurdy, Smith Giri, Shakira Grant, Bram Rochwerg, Erica Winks, Ashley E. Rosko, Monika Engelhardt, Charlotte Pawlyn, Gordon Cook, Graham Jackson, Sara Bringhen, Thierry Facon, Alessandra Larocca, Sonja Zweegman, Tanya M. Wildes

**Affiliations:** 1 Department of Oncology, McMaster University, Hamilton, ON Canada; 2 Department of Medicine, The Ottawa Hospital, Ottawa, ON Canada; 3 Department of Medicine, University of Alabama at Birmingham, Birmingham, AL USA; 4 Department of Medicine, Division of Hematology, The University of North Carolina at Chapel Hill, Chapel Hill, NC USA; 5 Department of Medicine, McMaster University, Hamilton, ON Canada; 6 Department of Health Research Methods, Evidence and Impact, McMaster University, Hamilton, ON Canada; 7 Division of Hematology, The Ohio State University, Columbus, OH USA; 8 Hematology and Oncology Department, Interdisciplinary Cancer Center (ITZ) and Comprehensive Cancer Center Freiburg (CCCF), Faculty of Freidburg, University of Freiburg, Hugstetterstr. 53, 79106 Freiburg, Germany; 9 The Institute of Cancer Research and Royal Marsden Hospital NHS Foundation Trust, London, UK; 10 The Royal Marsden Hospital NHS Foundation Trust, London, UK; 11 Cancer Research UK Clinical Trials Unit, LICTR, University of Leeds, Leeds, UK; 12 Northern Centre for Cancer Care, Newcastle Upon Tyne Hospitals Trust, Newcastle Upon Tyne, UK; 13 SSD Clinical Trial in Onco-hematology and Multiple Myeloma, AOU City of Health and Science of Turin, Torino, Italy; 14 University of Lille, CHU Lille, Service des Maladies du Sang, Lille, France; 15 French Academy of Medicine, Paris, France; 16 SSD Clinical trials in onco-ematologia e mieloma multiplo, Division of Hematology, University of Torino, Azienda Ospedaliero-Universitaria Città della Salute e della Scienza di Torino, Torino, Italy; 17 Department of Hematology, Amsterdam UMC, Vrije Universiteit Amsterdam, Cancer Center Amsterdam, Amsterdam, the Netherlands; 18 Division of Hematology/Oncology, University of Nebraska Medical Center, Omaha, NE USA

**Keywords:** Myeloma, Medical research

## Abstract

Multiple myeloma (MM) is an incurable blood cancer that primarily affects older adults. Several frailty tools have been developed to address the heterogeneity of aging in this population. Uptake of these measures has been variable, leading to a gap in knowledge regarding the proportion of enrolled trial participants considered frail and uncertainty in the treatment-related effects and outcomes among this high-risk population. We performed a systematic review of therapeutic interventional MM clinical trials reporting on frailty. We included 43 clinical trials (24 randomized controlled trials and 19 non-randomized trials) which met eligibility criteria. Frailty was increasingly incorporated in studies in more recent years with 41.9% of included studies being reported in the last two years. Commonly used frailty tools included the International Myeloma Working Group (IMWG) frailty index (41.8%), and the simplified frailty score (39.5%). Frailty status was categorized with 3 levels as (frail, intermediate fit, or fit) in 51.2% of the studies and dichotomized (frail, non-frail) in 18.6% of studies. Frailty prevalence greatly varied across trials ranging from 17.2% to 73.6% of the cohort. Of the included studies, 72.0% conducted subgroup analysis (planned or post-hoc) based on frailty status. Most studies demonstrated a consistent benefit of MM interventions among the frail and non-frail populations, however in general, frail patients had worse outcomes compared to the fit. Although frailty is increasingly being incorporated in MM clinical trials, due to the variation in both the definition and categorization of frailty, there remains heterogeneity in the prevalence of frailty and its potential associated impact on outcomes.

## Introduction

Multiple myeloma (MM) is an incurable plasma cell neoplasm associated with significant morbidity and mortality. It is considered a disease of older adults, with a median age at diagnosis of 69 years [1]. Despite survival gains for patients with MM over the past 2 decades, including advances in available therapeutic agents, outcomes of older adults still lag behind [2]. Older adults represent a heterogeneous group with wide variations in functional status and overall disease-related outcomes [3]. Incorporating frailty assessments can help improve the current understanding of the heterogeneity of aging in various disease states, including MM. Frailty is defined as a state of vulnerability to adverse health outcomes when exposed to an external stressor [4, 5]. Although frailty is age-related, advanced chronological age does not equate to frailty, creating heterogeneity in the aging process.

Several tools have been developed to assess frailty [6], yet operationalizing frailty in clinical practice remains challenging. Of the existing frailty measures used in geriatrics, the most well-known are the Fried frailty phenotype [7] and the deficits accumulation model [8]. Studies have since sought to simplify frailty measures and apply them to select populations with cancer. Among patients with MM, two common tools include the International Myeloma Working Group (IMWG) frailty score [9] [incorporates chronological age, Charlson co-morbidity index, activity of daily living (ADLs), independent activity of daily living (IADLs)] and the simplified frailty score (modified IMWG frailty score) by Facon et al. [10] (incorporates age, ECOG performance status and Charlson co-morbidity index). In subsequent studies incorporating these scores, 33%-50% of older adults with transplant-ineligible MM are classified as frail [11]. Patients classified as frail have worse progression-free and overall survival, and increased rates of infection, treatment toxicity, and chemotherapy discontinuation rates for frail older adults compared to fit individuals [9, 12].

Given the importance of frailty in understanding outcomes in MM, clinical trials have recently started incorporating frailty assessment into their data collection. Some studies incorporating frailty measures have used fitness-based approaches to assign therapies or conducted posthoc subgroup analyes [13–15]. However, overall uptake of these frailty measures across clinical trials has been variable, leading to a gap in knowledge regarding the proportion of enrolled trial participants considered as frail and uncertainty in frailty-related treatment effects and outcomes. Understanding the definition and subsequently the prevalence of frailty and its impact on outcomes represents an important step in devising future targeted therapies to optimize outcomes in this high-risk MM subgroup.

To our knowledge, no prior systematic review has been conducted to assess the impact of frailty on treatment outcomes in therapeutic MM trials. Therefore, the objective of this systematic review was to 1) examine prevalence of frailty in therapeutic MM trials and 2) evaluate outcomes among frail older adults in MM clinical trials.

## Methods

There was no external funding for this review. We registered this systematic review on PROSPERO (#CRD42022324068) and report the results according to the Preferred Reporting Items for Systematic Reviews and Meta-analyses (PRISMA) guidelines [16].

### Search strategy

We created and conducted the search strategy with input from all the authors and a medical librarian (E.U. from the E.M. Uleryk Consulting). We searched the following databases from inception to April 5, 2022: MEDLINE and Embase (OvidSP); Scopus (Elsevier); Web of Science (Clarivate), and Cochrane Library (Wiley). We used a combination of controlled vocabulary (MeSH [Medical Subject Headings] and Emtree terms] and keywords with various synonyms for the following concepts: “multiple myeloma” AND (“frailty” or “geriatric assessment”). We limited the search strategy to English language studies. The full search strategy for each database is available in Supplementary Table S1.

We also did a manual search of (1) bibliographies of any included trials or relevant review articles, (2) ongoing clinical trials (clinicaltrial.gov), and (3) conference abstracts (from 2015-2021 for the American Society of Hematology and from 2015-2022 for the American Society of Clinical Oncology and the European Hematology Association). We imported citations from all databases into an EndNote X9 database. After removing duplicate articles, two independent reviewers (H.M. and A.M.) screened the remaining citations. We included the most recent analysis if studies had multiple interim analyses or abstracts. The same two team members (H.M and A.M) reviewed full text to confirm eligibility for any citation deemed potentially relevant. If there were disagreements, the article was reviewed by a third reviewer (T.W).

### Selection criteria

We used the following eligibility criteria for included studies: (1) included an evaluation of therapeutic drug agent for newly diagnosed (NDMM), or relapsed/refractory (R/R) patients (2) was a clinical trial (phase I to IV, we excluded real-world observational cohort and registry database studies) (3) reported on a measure of frailty (intermediate fit or frail) either as inclusion criteria for trial entry, baseline characteristics or post-hoc analysis. We defined frailty measures as any screening or comprehensive geriatric assessment tools which included ≥2 aging-associated domain assessments. These domain assessments could include a combination of age, co-morbidities, functional/performance status [17]. We excluded any study that classified frailty based solely on one factor alone (i.e., studies categorizing patients as being frail solely based upon age, eastern cooperative oncology group performance status (ECOG PS), Karnofsky performance status (KPS), or comorbidities alone). We excluded studies that did not indicate how frailty was defined, as it was not possible to ascertain if ≥2 aging-associated domains were included.

### Data extraction

We extracted study data, including first author, years of trial enrollment, trial phase (I, II, or III/IV), study methodology (randomized controlled trial [RCT] vs. non-RCT), disease phase (NDMM vs. R/R), sample size, trial location, and therapeutic agents in both the experimental and control arms. The assessment tool utilized for frailty assessment was recorded. A study was recorded as using the IMWG frailty score if age, Charlson co-morbidity index, ADLs and IADLs were assessed or if the study self-reported as using IMWG frailty Index [9]. A study was recorded as using the simplified frailty score (also known as modified IMWG frailty index) if ECOG PS was used instead of ADLs and IADLs [10]. We also recorded the frailty categorization (two or three subgroups), frailty prevalence, patient characteristics (median age) and outcome data (efficacy and toxicity data).

### Definition of outcomes

Progression-free survival (PFS) was defined in all studies as the time from randomization to the disease progression or death, whichever came first. Additional outcomes recorded included overall survival (OS), overall response rates (ORR) [18], and ≥ grade 3 treatment-emergent adverse events (TEAEs). We also included quality of life or patient-reported outcomes stratified by frailty, if available. For the outcomes of PFS and OS, we extracted hazard ratios and 95% confidence intervals whenever available.

## Results

**Fig. 1:**
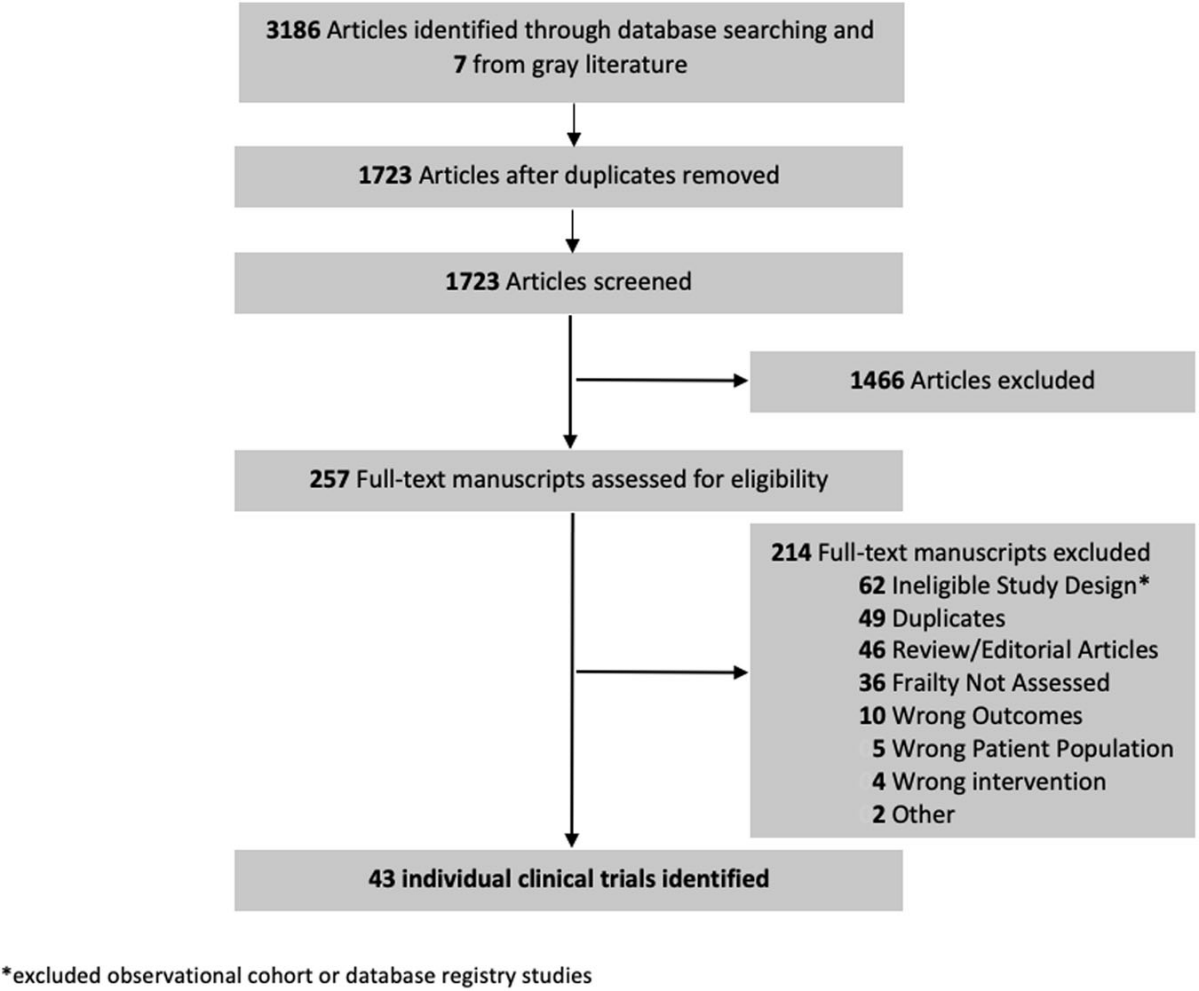
Flow Diagram of Study Selection. The process of selected the included studies is indicated.

Of 3193 studies, we included 257 in the full-text review (Fig. 1). After a full-text review, 43 clinical trials met the eligibility criteria for inclusion in this review. Common reason for exclusion during the full-text review included ineligible study design such as observational cohort or database registry studies (62/257, 24.2%) and not assessing or reporting on frailty (36/257, 14.1%).

### Study characteristics

**Fig. 2:**
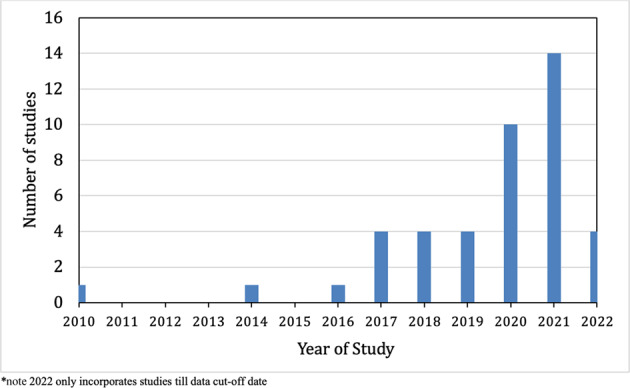
Number of studies evaluating or reporting frailty assessments each year.

Summary characteristics of the 43 included studies are presented in Table 1. This included 24 RCTs and 19 non-randomized trials. A total of 26/43 (60.4%) and 17/43 (39.5%) of the studies were in the NDMM and R/R settings, respectively. Most studies were multicenter (38/43, 88.3%), with a plurality conducted in Europe (20/43, 46.5%). There were increasing number of studies evaluating or reporting on frailty in more recent years with 18/43 (41.9%) in the last two years (Fig. 2).

**Table 1:** Summary characteristics of the included studies evaluating frailty in MM therapeutic clinical trials.

	*N* = 43
**MM disease phase**	
Newly-diagnosed	26 (60.4%)
Relapsed/Refractory	17 (39.5%)
**Study Methodology**	
Randomized controlled clinical trial	24 (55.8%)
Non-Randomized controlled clinical trial	19 (44.2%)
**Clinical Trial Phase**	
Phase I/II	20 (46.5%)
Phase III/IV	18 (41.9%)
Unknown	5 (11.6%)
**Multicentre**	
Yes	38 (88.3%)
No	5 (11.6%)
**Geographic Region of Study**	
Europe	20 (46.5%)
Global	11 (25.6%)
Asia	7 (16.3%)
United States	3 (7.0%)
Other	2 (4.6%)
**Reported within last two years (2021, 2022)**	18 (41.9%)
Reason for Frailty Evaluation*	
Subgroup analysis	31 (72.0%)
Study entry criteria	8 (18.6%)
Intervention based upon frailty	1 (2.3%)
Drug dosing	5 (11.6%)
Longitudinal assessment (> 1 time point)	4 (9.3%)
Tools Utilized for Frailty Assessment*	
International Myeloma Working Group Index	18 (41.8%)
Simplified frailty score	17 (39.5%)
Other or unknown	14 (32.6%)
**Frailty Categorization**	
Three (fit-intermediate fit-frail)	22 (51.2%)
Two (fit-frail)	8 (18.6%)
Continuous	1 (2.3%)
Not available or not applicable	12 (27.9%)

*A study could be categorized into more than category

Further study characteristics for the 24 RCTs (16 in NDMM and 8 in R/R) are shown in Table 2. The median age of the patients ranged from 73 to 77 in the NDMM trials and 64 to 70 years in the R/R setting. Among the included RCTs, planned sample sizes ranging from *N* = 112 (Muk eight [19]) to *N* = 1852 (Myeloma XI [20]). Nineteen non-randomized studies (10 in NDMM and 9 in R/R) were included (Table 3). The median age of patients in these studies ranged from 62 to 82 years. These studies varied, including small single-center studies with *n* < 20 (3/19, 15.8%) [21–23] to a larger phase II study with 238 participants (HOVON 123 [24]).

**Table 2:** Randomized controlled trial of therapeutic agents in multiple myeloma incorporating or reporting on frailty.

	Study Name	Intervention arm	Control arm	Median age	Frailty definition	Frailty categories	Frailty prevalence	Outcomes for frail subgroup (intervention vs control arm)*
**Newly-Diagnosed Multiple Myeloma**								
1	Facon et al. (2022) MAIA [31] *N* = 737, Global Phase III	Dara/Len/Dex	Len/Dex	73	Simplified frailty score	Subgroup included: post-hoc, fit, intermediate fit, frail^#^	341 (46.3%)	PFS (NR vs 30.4, HR 0.62, *p* = 0.003) OS: Not available ORR (87.2% vs 78.1%; *p* = 0.0265) Grade ≥ 3 TEAE (94.6% and 89.2%)
2	Larocca et al. (2021) *N* = 199, Europe Phase III [25]	Len/Dex X 9 followed by reduced Len	Len/Dex	76	IMWG	Study entry criteria: Intermediate fit	Not applicable	PFS (20.2 vs 18.3, HR, 0.78, *p* = 0.16) OS 3-yr (74% vs 63%, *p* = 0.06) ORR (78% vs 68%, *p* = 0.15) ≥ 1 non-hem Grade ≥ 3 AE (33% vs 43%; *P* = 0.14)
3	Jackson et al. (2021) Myeloma XI *N* = 1852, Europe Phase III [20]	Cyclo/Len/Dex X 6-8 followed by maintenance randomization	Cyclo/Thal/Dex X 6-8 followed by maintenance randomization	74	UK MM Research Alliance Risk Profile tested and validated	Subgroup included: Post-hoc, Low, medium, high risk	High risk: 617 (33.3%)	PFS (12 vs 12, HR 0.98, *p* = 0.830) OS (31 vs 24, HR 0.89, *p* = 0.224) ORR NA Toxicity NA
4	Mateos et al. (2021) ALCYONE *N* = 706, Global Phase III [32]	Dara/Bort/Mel/Pred X 9 followed by dara maintenance	Bort/Mel/Pred X 9	74	Simplified frailty score	Subgroup included: Post-hoc: fit, intermediate fit, frail^#^	315 (44.6%)	PFS (32.9 vs 19.5, HR 0.51, *p* < 0.0001) OS 36 m (71.4% vs 59.0%, HR 0.66, *p* = 0.0292) ORR (88.3% vs. 72.4%, *p* = 0.0003) Grade ≥ 3 TEAE (79.4% vs 81.5%, *p* = N/A)
5	Mina et al. (2021) EMN10 Unito *N* = 171, Europe, Phase II [26, 50]	1.Ixa/Cyclo/ex 2.Ixa/hal/dex 3.Ixa/benda/ex X 9 followed by Ixa maintenance	Ixa/dex X 9 followed by Ixa maintenance	74	IMWG	Subgroup included: Post hoc analysis, fit, intermediate fit, frail	43 (25.1%)	Data for interventional vs control not available for frail subgroup
6	O’Donnell et al. (2021) Ongoing *N* = 188 target, USA AFT-41 Phase II [41]	Len/Ixa/Dara/Dex X 12 followed by Len	Len/Ixa/Dara/Dex X 12 followed by Len/Ixa/Dara	Ongoing	Alliance GA	Subgroup included: prospective, categories unknown	Not available	Not available
7	Cook et al. (2021) Ongoing, UKMRA FiTNEss (N-180/740), Europe, Phase III [27]	Adaptive (IMWG frailty adjusted dosing) Ixa/Len/Dex	Standard (reactive dosing) Ixa/Len/Dex	Ongoing, 77	UK MM Research Alliance Risk Profile, IMWG	Frailty adapted design/longitudinal: Prospective, Fit, unfit, frail Or low, medium, high	IMWG frail 84/180 (46.7%) UK MM risk profile 75/180 (41.7%)	Not available
8	Zweegman et al. (2020) HOVON 126 *N* = 143, Europe, Phase II [33]	Ixa/Thal/Dex x 9 followed by Ixa maintenance	Ixa/Thal/Dex x 9 followed by placebo maintenance	73	Simplified frailty score^	Subgroups included: fit, intermediate fit, frail	63 (44.8%)	Not available
9	Facon et al. (2020) FIRST *N* = 1623, Global, Phase III [10]	1. Len/Dex X 18 2. Len/Dex	Mel-Thal-Pred X 12	73	Simplified frailty score derived and validated using this cohort	Subgroup included: Post-hoc, Nonfrail vs frail	790 (48.6%)	Len/Dex vs Mel/Thal/pred PFS (19.4 vs 19.0, HR 0.75, *p* = 0.005) OS (44.3 vs 38.5, HR = 0.84; *P* = 0.11) ORR Not available Grade ≥ 3 TEAE (HR 1.03, *p* = 0.796)
10	Bringhen et al. (2020) MM4 *N* = 706, Global, Phase III [28]	Induction-Ixa maintenance	Induction-placebo maintenance	73	IMWG	Subgroup included: fit, unfit, or frail	170 (24.1%)	PFS (15.4 vs 11.1, HR 0.733, *p* = 0.147) OS Not available ORR Not available Grade ≥ 3 TEAE (19% vs 9%, *p* = not available)
11	Brioli et al. (2020) GERMAIN *N* = 85, Europe, halted poor accrual Phase IIB [51]	Bort-Mel-Pred X 9 followed by Len maintenance	Bort-Mel-Pred followed by observation	75	Modified IMWG (EQ5D used to estimate ADLs and IADLs)	Subgroup included: retrospective fit, intermediate fit, or frail	45 (54.0%)	Data for interventional vs control not available for frail subgroup specifically
12	Bringhen et al. (2020) EMN01 *N* = 662, Europe Phase III [29]	1. Mel/Len/Pred 2. Cyclo/Len/Pred X 9 Followed by randomization to Len or Len/Pred	Len/Dex X 9 followed by randomization to Len or Len/Pred	73	IMWG	Subgroup included: post-hoc analysis fit, intermediate fit or frail	165 (24.9%)	Induction: PFS (21.5/13.8 MPR/CPR vs 18.2 Rd, p=NS) OS (44.7/40.5 MPR/CPR vs 48.2 Rd, p=NS) Grade 3 Non heme (all induction): 42% Maintenance: PFS (RP vs R HR 0.90, *p* = 0.67) OS (RP vs R, HR 1.04, *p* = 0.89) Grade ≥ 3 Non-heme (all maintenance): 13%
13	FRAIL-M; Spencer, Andre (2019), ongoing, *N* = 69/300 enrolled, Australia and New Zealand, Phase II [60]	1.Bort/Len/Dex 2. Bort/Dex	Len/Dex	Not available	Unknown	Subgroup included: primary outcome defined by frailty including ORR, toxicity fit, intermediate fit, frail	Not available	Not available
14	PI: Facon (2019) IFM 2017-03 *N* = 294 target, active not recruiting, Europe, Phase III [30]	Len/Dara subq	Len/Dex	Not available	IMWG, Simplified Frailty score	Study entry inclusion criteria: frail (score ≥ 2)	Not applicable	Not available
15	PI: Larocca (2017), ongoing, target *N* = 350, Europe, Phase IV [42]	Bort/Mel/Pred X 9	Len/Dex	Not available	A frailty score based on age, comorbidities, physical and cognitive functioning	Subgroups included: planned secondary outcome, fit, intermediate fit, frail	Not available	Not available
16	Stege et al. (2017), HOVON-87, *N* = 637, Europe, Phase III [34]	Mel/Len/Pred X 9 followed by Len maintenance	Mel/Thal/Pred X 9 followed by Thal maintenance	73	Simplified frailty score^	Subgroup included: fit, intermediate fit, or frail	259 (40.7%)	Not available
**Relapsed/Refractory**								
17	Rocafiguera et al. (2022) OPTIMISMM (*N* = 559), Global, Phase III [48]	Pom/Bort/Dex	Bort/Dex	68	Simplified frailty score	Subgroup included: post hoc analysis non-frail or frail	186 (33.2%)	PFS (9.7 vs 5.1, *p* = 0.006) OS Not available ORR (79.6% vs 41.9%, *p* < 0.001) Grade ≥ 3 TEAE (96.8 vs 87.9%, *p* = not available)
18	Quach et al. (2022) CANDOR (*N* = 446), Global, Phase III [47]	Dara/Car/Dex	Car/Dex	64	Simplified frailty score^	Subgroup included: post hoc analysis fit, intermediate fit or frail	118 (26.5%)	PFS (18.5 vs 9.3, HR 0.66, 95% CI 0.38–1.14) OS ORR 75% vs 54% (OR 2.39, 95% CI 1.09–5.22) Grade ≥ 3 TEAE 91% and 90%, *p* = not available)
19	Auner et al. (2022) Muk eight *N* = 112, Europe, Phase II [19]	Ixa/Cyclo/Dex	Cyclo/Dex	70	Simplified frailty score	Subgroup included: post-hoc analysis non-frail, frail	81 (73.6)%	PFS (6.7 vs 5.6, HR 1.05, 80% CI 0.78-1.40) OS (14.1 vs 18.0, HR 1.49, 80% CI 0.99-2.23
20	Auner et al. (2021) BOSTON *N* = 402, Global, Phase III [44]	Seli/Bort/Dex	Bort/Dex	66	Simplified frailty score	Subgroup included: post-hoc analysis non-frail, frail	130 (32.3%)	PFS (13.93 vs 9.46, HR 0.69, *p* = 0.081) OS (NR vs 23.49, HR 0.62, *p* = 0.061) ORR (69.7% vs 60.9%, *p* = 0.148) Serious TEAE (59.1 % vs 48.4%)
21	Schjesvold et al. (2021) ICARIA *N* = 307, Global, Phase III [45]	Isa/Pom/Dex	Pom/Dex	70	Simplified frailty score	Subgroup included: post-hoc analysis Fit/intermediate fit vs frail	86 (28.0%)	PFS (9.0 vs 4.5, HR 0.81, pvalue=0.493) OS 1 yr (66.9% vs 58.5%) ORR (52.1% vs 34.2%, *p* = 0.048) Grade [31] 3 TEAE (91.7% vs 80.6%)
22	Facon et al. (2020) ASPIRE *N* = 792, Global, Phase III [46]	Carf/Len/Dex	Len/Dex	64	Simplified frailty score^	Subgroup included: post-hoc analysis fit, intermediate fit, frail	196 (24.7%)	PFS (24.1 vs 15.9, HR 0.78, *p* = 0.085) OS (36.4 vs 26.2, HR 0.79, *p* = 0.070) ORR (84% vs 64%, *p* = N/A) Grade ≥ 3 TEAE (93% vs 94%)
23	Facon et al. (2020) ENDEAVOR *N* = 929, Global Phase III [46]	Carf/Dex	Bort/Dex	65	Simplified frailty score^	Subgroup included: post-hoc analysis fit, intermediate fit, frail	330 (35.5%)	PFS (18.7 vs 6.6, HR 0.50, *p* = < 0.01) OS (33.6 vs 21.8, HR 0.75, *p* = 0.026) ORR (76% vs 54%, *p* = N/A) Grade ≥ 3 TEAE (85% vs 79%)
24	Facon et al. (2020) ARROW *N* = 478, Global, Phase III [46]	Carf/Dex 70 mg/m^2^	Carf/Dex 27 mg/m^2^	66	Simplified frailty score^	Subgroup included: post-hoc analysis fit, intermediate fit, frail	141 (29.5%)	PFS (10.3 vs 6.6, HR 0.76, *p* = 0.098) OS not available ORR (56% vs 41%, *p* = N/A) Grade ≥ 3 TEAE (81% vs 70%)

*median PFS and OS in months unless otherwise indicated, toxicity was included if information was available. *Bort* Bortezomib, *Carf* Carfilzomib, *Clairtho* Clarithyromycin, *Cyclo* Cyclophosphamide, *Dara* Daratumumab, *Dex* Dexamethasone, *Ixa* Ixazomib, *Len* Lenalidomide, *lipDox* Liposomal doxorubicin, *Mel* Melphalan, *Pom* Pomalidomide, *Pred* Prednisone, *Thal* Thalidomide, *PR* Partial response, *IMWG* International Myeloma Working Group, *PFS* Progression free survival, *OS* Overall survival, *ORR* Overall response rate, *TEAE* Treatment emergent adverse event ^^^Simplified frailty score calculated based upon age, CCI and ECOG PS, but reported as three IMWG modified categories ^#^also classified and reported outcomes based upon two groups non frail (fit + intermediate) and frail

**Table 3:** Non-randomized controlled trial of therapeutic agents in multiple myeloma incorporating or reporting on frailty.

	Study Name	Setting	Therapeutic agents	Median age	Frailty definition	Frailty categories	Frailty prevalence	Frailty based outcomes*
**Newly-diagnosed MM**								
1	Bao et al. (2021) [43]	Multicentre, China, (*N* = 95/120), ongoing, phase unknown	Two arms: Ixa/Lipodox/Dex or Ixa/Len/Dex If ≥ PR, Ixa/Dex maintenance	71	Study entry criteria: frail as per IMWG or Mayo geriatric vulnerability scoring system	Not applicable	Not applicable	IAd vs IRd PFS 16 vs NR OS NR vs NR ORR 80.9% vs 81.0% ≥ Grade 3 heme tox: 13.7%
2	Nakazato et al. (2021) [40]	Multicenter, Japan, phase II (*N* = 47)	Bort/Cyclo/Dex X 4 followed by Bort/Thal/Dex X 4 (dose for frailty)	75	Drug dosing: Vulnerable Elders Survey (VES-13)	Fit (VES-13 < 3), Frail (≥ 3)	31 (66.0%)	3 yr PFS 53.3% 3 yr OS 77.6% ORR 87.1%
3	Stege et al. (2021) HOVON 143 [49, 61]	Multicentre, Europe, phase II (*N* = 65)	Ixa/Dara/Dex X 9 followed by Ixa/Dara for 2 years	81	Study entry criteria: IMWG frail pts, IMWG intermediate fit patients, longitudinal assessment	Not applicable	Not applicable	PFS 13.8 1 year OS 78% ORR 78% (induction) ≥ Grade 3 heme tox 31% ≥ Grade 3 non-heme 74%
4	Stege et al. (2021, 2018) HOVON 123 [24, 54]	Multicentre, Europe, phase II, (*N* = 238)	Bort/Mel/Pred X 9	77 unfit 81 frail	Subgroup included: IMWG frailty score plus other GA parameters; longitudinal assessment	Fit, unfit, frail	61.0%	OS 31 Tx discontinuation 51% Unfit superior QoL than frail
5	PI: Davis, Tyler (2020) Actively Recruiting (MMY2035) [53]	Multicentre, USA (*N* = 44) phase II	Dara/Len/Dex	Not available	Study entry criteria: IMWG intermediate fit and frail Drug dosing: adjusted for frailty	Not applicable	Not available	Not available
6	PI: Cohen, Yael (2019) Recruitment completed [62]	Multicentre, Israel (*N* = 41), phase II	Carf/Dara/Len/Dex	Not available	Subgroup included: fit, intermediate fit, frail as per IMWG Drug dosing: adjusted for frailty	Fit, intermediate fit, frail	Not available	Not available
7	Larocca et al. (2018) [52]	Multicentre, Europe, (*N* = 58), phase II	Carf/Cyclo/Dex X 9 followed by Carf maintenance	71	Subgroup included: IMWG frailty score	Fit, unfit, Frail	10 (17.2%)	Not available
8	Tuchman S et al. (2017) [21] VCD lite	Single centre, United States, phase II, *N* = 14 (closed due to slow accrual)	Bort/Cyclo/Dex followed by bort/len maintenance	76.5	Subgroup included: Cancer and Aging Research Group Geriatric Assessment	Risk score from 0-19	Median score 10	Not applicable as continuous score
9	PI: Yoshihara, Satoshi (2017) Recruitment pending [63]	Single centre, Japan, (*N* = 30 target), Phase unknown	-Dara/Len/Bort/Dex (Ixa/len/dex if not tolerable) X 12 followed by maintenance	Not available	Drug dosing: IMWG adjusted for frailty	Not available	Not available	Not available
10	Larocca et al. (2016) [64]	Multicentre, Italy (*N* = 152), Phase II	Sequential 1.Bor/Mel/Pred 2. Bor/Cyclo/Dex 3. Bort/Pred	78	Subgroup included: IMWG	Fit, unfit, frail	82 (54%)	PFS 13.8 months 2 year OS 60% ORR 65% Drug related SAE ≥ 13%
**Relapsed/Refractory**								
11	PI: Touzeau, Cyrille (2021) recruiting, IFM 2021_03 [35]	Multicentre, Europe, (*N* = 80 target), Phase II	Ixa/Iberomide/Dex	Not available	Subgroup included: simplified frailty score	Not available	Not available	Not available
12	Macro et al. (2021) IFM 2018-02 [36]	Multicentre, Europe, phase II (*N* = 44/50 enrolled)	Ixa/dara/methylpred	82	Study entry criteria: frail as per IMWG and simplified frailty score	Not applicable	Not applicable	PFS not available OS not available ORR 86% in those continuing tx and 71% stopped tx
13	Lee et al. (2020) KMMWP-164 [37]	Multicentre, South Korea, phase II (*N* = 55)	Pom/Cyclo/Dex	74	Subgroup included: Simplified frailty score	Nonfrail, frail	31 (56.4%)	PFS 7.36 months OS 18.48 months
14	PI: Ho Sup Lee, Kosin (2019) Recruiting [39]	Multicentre, Korea, Phase II (*N* = 102)	Len/Dex	Not available	Study entry criteria: R-MCI, Inadequate (intermediate), frail	Not applicable	Not available	Not available
15	Waldschmidt et al. (2018) VERUMM [38]	Single centre, Germany Phase I/II (*N* = 33)	Bort/Dox/Dex/Vorinostat	62	Subgroup included, Longitudinal assessment: R-MCI, IMWG, Kaplan Feinstein Index, Timed up and go, cognitive testing	Not available	Not available	Subset of frailty score improved from baseline to end of treatment
16	Ludwig et al. [65]. (2018)	Multicentre, Europe, Phase II (*N* = 90)	Ixa/Thal/Dex X 8 followed by Ixa maintenance	67.3	Subgroup included: Secondary endpoint IMWG frailty index	Fit, unfit, frail	Not available	PFS 10.9 OS Not reached
17	PI: Unknown (2018) Active, not recruiting [66]	Multicentre, China, (*n* = 30), phase unknown	Ixazomib	Not available	Subgroup included/secondary outcome, IMWG	Fit, unfit, frail	Not available	Not available
18	Nakazato et al. (2014) Recruitment status unknown Personalized BiRd [23]	Single Centre, Japan (*n* = 20), phase unknown	Clarithro/Len/Dex Len dose adjusted according to frailty	Not available	Drug dosing: according to VES-13	Fit (VES-13 < 3) and Frail (VES-13 ( ≥ 3)	Not available	Not available
19	Mele et al. (2010) [22]	Single centre, Italy (*N* = 18), phase unknown	Bort/Cyclo/Dex	76	Study entry criteria: ≥ 1 geriatric syndromes, or more moderate–severe comorbidities, and (WHO) PS > 2	Not applicable	Not available	Not available

*median PFS and OS in months unless otherwise indicated, toxicity was included if information was available; if studies contained intermediate fit and frail patients, outcomes are only described for frail patients. *Bort* Bortezomib, *Clarithro* Clarithyromycin, *Cyclo* Cyclophosphamide, *Dara* Daratumumab, *dex* Dexamethasone, *Ixa* Lxazomib, *Len* Lenalidomide, *lipDox* Liposomal doxorubicin, *Mel* Melphalan, *Pom* Pomalidomide, *Pred* Prednisone, *Thal* Thalidomide, *PR* Partial response, *IMWG* International Myeloma Working Group, *PFS* Progression free survival, *OS* Overall survival, *ORR* Overall response rate, *VES-13* Vulnerable elders survey, *NR* Not reached.

### Frailty measurement tools

The most commonly used tool for frailty assessment was the IMWG frailty score (18/43, 41.8%). Among the RCTs, IMWG frailty score was used or is currently being used in a total of 6 NDMM studies (Larocca et al. [25], EMN10 [26], UK FiTNEss [27], MM4 [28], EMN01 [29], IFM 2017_03 [30]). Among the non-RCTs, the IMWG frailty score was utilized in 12 studies (8 NDMM and 4 R/R). The simplified frailty score was the next most commonly utilized score (17/43, 39.2%). Among the RCTs it was used in 6 NDMM studies (MAIA [31], ALCYONE [32], HOVON 126 [33], FIRST [10], HOVON-87 [34], IFM 2017_03 [30]) and all of the 8 studies RCT in the R/R setting. Among the non-RCT, it is currently being utilized in 3 studies in the R/R setting (IFM2021_03 [35], IFM 2018_02 [36] and KMMWP-164 [37]). The Revised Myeloma Comorbidity Index was used in two studies [38, 39]. Other studies incorporated non-MM-specific geriatric assessment tools, including the VES-13 [23, 40], CARG geriatric assessment [21], Alliance geriatric assessment tool [41], or other geriatric domains (two or more components of geriatric assessment, including comorbidities, cognition, and functional/physical assessments) [22, 24, 38, 42, 43].

### Reason for frailty assessment in the trial

Frailty assessment was conducted as a subgroup analysis (planned or post-hoc) in 31/43 (72.0%), for study entry criteria in 8/43 (18.6%), or for drug dosing in 5/43 (11.6%) of the included studies. These included studies both in the NDMM setting (the large phase III trials MAIA [31] and ALCYONE [32]) as well as studies in the R/R setting (MUK eight [19], BOSTON [44], ICARIA [45], ASPIRE, ENDEAVOR, ARROW [46] and more recently CANDOR [47] and OPTIMISMM [48]). Two RCTs evaluated frailty specifically for study entry (Larocca et al. enrolled intermediate fit patients only [25] and the ongoing study IFM 2017-03 [30], an RCT specifically designed for frail patients). The UKMRA FiTNEss study [27], an ongoing phase III RCT, was the only study that used frailty to guide treatment delivery into its primary trial design. With regards to longitudinal changes, one prior study (VBDD-VERRUM) [38] evaluated how frailty changed longitudinally over time in R/R MM. This will be further studied in the HOVON 123 [24] and 143 [49] studies along with the UKMRA FiTNEss [27]study will evaluate the dynamic nature of the IMWG frailty index in the longitudinal setting.

### Frailty categorization and prevalence

Frailty categorization varied across the different studies, with dichotomous, ordinal or continuous reporting being used. Frailty was divided into three levels (frail, intermediate fit, fit) in 22/43 (51.2%) of the studies and dichotomized (frail, non-frail) in 8/43 (18.6%). Continuous categorization of frailty was present in one study which used the Cancer and Aging Research Group Geriatric Assessment [21].

Given the varied categorization of frailty in either two or three subgroups, frailty prevalence varied greatly across studies. In the RCTs, in the NDMM studies, frailty prevalence ranged from 25.1% [50] to 54.0% [51]. In the R/R setting, many RCTs reported frailty prevalence as high as 73.6% as in the trial Muk eight [19]. Among the non-RCTs, frailty prevalence ranged from 17.2% [52] to 66.0% [40].

### Impact of frailty on disease efficacy outcomes

Disease-specific outcomes including PFS, OS, and ORR were reported in the majority of completed studies. In the RCT group, in the NDMM setting, several therapies were found to be beneficial in the frail subgroup, including the incorporation of anti-CD38 upfront. The ALCYONE and MAIA published post-hoc analysis using a simplified frailty score and demonstrated improvement for PFS with the addition of anti-CD38 also among frail older adults consistent with the overall trial results [31, 32]. In ALCYONE, the PFS benefit of daratumumab-bortezomib-melphalan-prednisone (D-VMP) versus bortezomib-melphalan-prednisone (VMP) for the frail population had a HR 0.51 (95% CI, 0.39-0.68) compared to the HR of 0.36 (95% CI, 0.28-0.47) for the total non-frail group (fit and intermediate fit) [32]. Similarly, in the MAIA trial the PFS benefit of daratumumab-lenalidomide-dexamethasone (D-Rd) versus lenalidomide-dexamethasone (Rd) for the frail population had a HR 0.62 (95% CI, 0.45–0.85) compared to the total non-frail population with a HR 0.48 (95% CI, 0.34–0.68) [31]. Overall, the magnitude of benefit with the addition of anti-CD38 was lower among the frail older adults as compared to the fit population in both trials and the addition of anti-CD38 did not overcome the negative impact of frailty.

Among RCTs in the R/R setting, although the point estimates for the efficacy outcomes were often improved in the frail subgroup in the interventional arm compared to the control arm similar to the overall trial population, the magnitude of benefit was attenuated and, in some cases, not statistically significant. In the ICARIA trial, for example, there was a benefit in PFS with isatuximab-pomalidomide-dexamethasone (IsaPd) as compared to pomalidomide-dexamethasone (Pd) in the fit/intermediate group with a HR of 0.49 (95% CI 0.33–0.73); however, this was less pronounced in the frail subgroup with a HR of only 0.81 (95% CI 0.45–1.48) [45]. This was consistent across a number of trials, including CANDOR [47], BOSTON [44], ASPIRE and ARROW [46] which all demonstrated improved outcomes with intervention among fit/intermediate fit patients, but with less pronounced benefit in the frail subgroup. Conversely, in the study MUK eight, which had a high proportion of patients classified as frail, the overall trial results were negative (no difference in primary outcome of progression-free survival of ixa-cyclo-dex compared to cyclo-dex), largely driven the by the impact of frailty on treatment delivery and overall regimen tolerability [19].

Among the non-RCTs, several studies specifically examining frail patients have been conducted or are ongoing. These include studies such as the HOVON 143 [49] in NDMM with an overall PFS of 13.8 months among patients being treated with ixazomib-daratumumab-dexamethasone followed by ixazomib-daratumumab maintenance for 2 years specifically in the subset of frail patients. Additional studies in NDMM include the ongoing MMY2035 study [53] which incorporates frailty-adjusted dosing for lenalidomide, with results expected in 2024. In the R/R setting, several studies are ongoing, including the IFM_2021_03 [35] and the IFM 2018_02 [36].

### Impact of frailty on toxicity outcomes

Toxicity outcomes specifically for the frail subgroups were reported in the majority of completed studies. Among the RCTs, toxicity was reported for contemporary trials, including ALCYONE and MAIA, in the NDMM setting. In the MAIA trial, higher rates of ≥ grade 3 TEAE events were observed with the addition of anti-CD38 in the frail subgroups, consistent with the overall trial population (94.6% DRd vs. 89.2% Rd in MAIA) [31]. In the frail subgroup in MAIA, there were of higher rates ≥ grade 3 neutropenia (57.7% DaraRd vs. 33.1% Rd) and infection (41.7% DaraRd vs. 27.7% Rd). Similarly, in the frail subgroup of the ALCYONE trial, higher rates ≥ grade 3 neutropenia (41.3% Dara-VMP vs. 34.4% VMP) and infection (30.0% Dara-VMP vs. 17.9% VMP) were observed with the addition of the anti-CD38 antibody [32]. In the R/R setting, there was increased toxicity seen with therapeutic interventions compared to the control group among the frail subgroup including in BOSTON [44], ICARIA [45], ASPIRE, ENDEAVOR, ARROW [46], CANDOR [47] and OPTIMISMM [48]. Furthermore, specific toxicities of agents such as ≥ grade 3 cardiac failure toxicity observed with carfilzomib was higher in frail patients compared to fit patients (KRd: fit 4% vs. frail 10%; Kd56mg/m^2^ fit 4% vs. 9% frail) across treatment groups [46].

Among the non-randomized RCTs, toxicity data and treatment discontinuation rates were available in only a subset of trials. HOVON 143, a phase II single-arm study conducted among patients classified as frail, reported high rates of non-hematological toxicity (74% of patients) [49]. This study also reported differences in outcomes among patients classified as frail based upon age alone or who were frail based upon additional geriatric impairments as defined by the IMWG frailty score (median PFS 21.6 months for patients who were frail based on age > 80 years alone versus 10.1 months in patients who were frail based age > 80 and additional geriatric impairments). HOVON 123, a phase II single-arm study, demonstrated higher treatment discontinuation rates among frail patients as compared to intermediate fit patients [24]. Furthermore, HOVON 123 was the only study available that reported quality of life and patient reported outcomes by frailty status, showing inferior quality of life among the frail patient group [54].

## Discussion

This analysis represents the first comprehensive systematic review evaluating the prevalence of frailty as well as the outcomes of frailty in MM therapeutic clinical trials. Frailty prevalence greatly varied across trials ranging from 17.2% to 73.6% of the cohort reflecting both differences in the populations as well as different measures of frailty.

Although it is encouraging that frailty is increasingly being incorporated in MM clinical trials, due to the wide variation in both the definition and categorization of frailty, there remains variation in which measure of frailty is used and heterogeneity in the prevalence of frailty, limiting evaluation of its potential impact on outcomes, as patients may be categorized differently in different frailty systems.

As the therapeutic treatment landscape of MM evolves, there is an increasing need to understand frailty as a means of identifying patients who may be at risk of not achieving the maximum benefit while also being at the highest risk of treatment toxicity. However, there remains a range of approaches to operationalizing the clinically intuitive concept of frailty making it challenging to evaluate both baseline populations as well as results across trials. Although the IMWG frailty scores is often thought of as the standard approach to defining frailty in MM [13], there was an increasing number of clinical trials in our review using the simplified frailty score. Although the simplified frailty score (comprising age, comorbidities and performance status alone) has facilitated retrospective post-hoc frailty analyses of previously conducted trials, it is important to note that this abbreviated score is both defined differently as well as often categorized differently compared to the IMWG frailty score. Furthermore, the simplified frailty score may not adequately encompass the heterogeneity in aging and may be more prone to subjective bias compared with prospective evaluation of frailty using more comprehensive tools, such as the IMWG, which incorporate functional status (activity of daily living and independent activity of daily living) [55]. The intermediate fit patients (IMWG frailty score), for example, in the study by Larocca et al. had a median PFS of 18.3 months with lenalidomide-dexamethasone; [25] whereas patient defined as fit/intermediate fit (simplified frailty score) in the MAIA trial had a median PFS of 41.7 months [31]. It is difficult to compare across trials; however, our study highlights that different definitions of this similar concept of frailty make this further challenging.

While the actual different ways of defining frailty make it challenging to compare across studies, the variation in categorizing a patient’s fitness status into either three (fit, intermediate fit, frail) or two (fit or frail) levels may further limit the ability to compare outcomes across different studies. This was further illustrated by Stege et al. where different weights for co-morbidities and cut off for frailty were utilized [56]. In this analysis done using the HOVON 123 study, revised frailty indices using different cut off was able to classify 45% fewer patients as frail, further improving the discriminative power of these scores. Even within sub-categories, there exists heterogeneity in outcomes as shown in the HOVON 143 study [49], where outcomes differed depending on which variable led to the frailty categorization (age and/or geriatric impairments). As frailty becomes increasingly incorporated into studies, clinicians will need to carefully evaluate both the frailty measure, categorization and the cut-off value used to define frailty across different studies.

The most common method for evaluating or conducting a frailty analysis in MM therapeutic trials was subgroup analyses. While many of the studies showed consistent improvements in outcomes with study interventions in frail subgroups, the magnitude of benefit was often less than those seen in fit patients. Furthermore, some of the studies, especially in the R/R setting, either showed no benefit or a substantially less benefit of the intervention with overall higher rates of toxicity. Given the often smaller and variable subset of frail older adults enrolled in these trials, it is not possible to exclude potential benefit from this high-risk subgroup. Larger studies with pre-specified subgroups that are adequately powered are needed to understand the potential benefit as well as toxicity of newer agents including bispecific antibodies and chimeric antigen receptor therapy in older adults with MM. Furthermore, clinical trials specifically focused on enrolling and optimizing therapeutic regimens for frail patients are needed further to improve outcomes in this clinical area of unmet need.

This review also highlights other key areas in incorporating frailty in MM therapeutic trials. While studies are increasingly reporting on frailty subgroups, incorporating frailty in primary study design for treatment delivery was uncommon, with only one study, the UKMRA FiTNESS study, incorporating frailty into the study design. To utilize frailty assessment to direct treatment delivery, rather than just describing the population, integration of frailty into primary study design will be pivotal for different phases of treatment, including NDMM and R/R disease. Another critical area is the need for studies to incorporate longitudinal frailty assessments. Longitudinal approaches to frailty may be important in lower treatment intensity for frail patients in the beginning, while potentially modifying treatment intensity as the frailty status changes. Unfortunately, existing frailty tools, including the IMWG frailty score, consist of largely static variables such as the categorical chronological age and pre-existing comorbidities and may be less suited for detecting changes in frailty over time. We do not yet know if frailty is modifiable or whether longitudinal changes in frailty will further optimize our treatment delivery; however, further development and validation of frailty assessment tools that are both sensitive, specific, and responsive to changes in frailty over time will be essential in the future evaluation.

The strength of this analysis includes the first comprehensive review highlighting the impact of frailty in therapeutic MM trials. We included randomized controlled trials and single-arm therapeutic MM trials. We used a comprehensive search and screening procedure with careful data abstraction. This review also has limitations. We only included studies registered as clinical trials with therapeutic drugs and therefore cannot report on the prevalence of frailty in the real-world which may be substantially higher given the exclusion of frail older adults often from clinical trials [57]. We also did not specifically include either in our search strategy or in evaluation of outcomes other biomarkers of frailty and/or sarcopenia which are known to impact clinical outcomes [58]. We also did not look at the relationship of frailty with other non-drug interventional studies such as physical activity, which are important components of overall MM management [59]. Lastly, given the heterogeneity in results and reporting, we could not conduct pooled analysis examining specific interventions and their efficacy or toxicity in frail compared to fit patients.

In conclusion, this systematic review summarizes how frailty is incorporated into therapeutic MM trials and highlights potential areas for future research. Although frailty assessments are being increasingly incorporated into trial designs, there remains wide heterogeneity in both the definition, categorization and cut-off for frailty among the different trials which may limit our ability to evaluate any associated outcomes. Future strategies aimed at standardizing frailty assessments, along with incorporation of frailty measures in the primary clinical trial design will be critical in operationalizing frailty and using fitness-based approaches to tailor the care of older frail older adults with MM.

## Supplementary information

Supplement

AJH checklist
