# Supplementary material for: The prevalence and outcomes of frail older adults in clinical trials in multiple myeloma: A systematic review
Source: Blood Cancer J. 2023 Jan 5;13(1):6. doi: 10.1038/s41408-022-00779-2 (PMC9813365; doi:10.1038/s41408-022-00779-2)
Supplement: Supplementary file 1 — Supplement [file 41408_2022_779_MOESM1_ESM.docx]

**Supplement**

**Table S1: Search strategy for included Databases**

*Ovid Medline, Medline Epub Ahead of Print, In-Process & Other Non-Indexed Citations, Daily (OvidSP)*

| **#** | **Searches** |
| --- | --- |
| 1 | multiple myeloma/ |
| 2 | (multiple adj2 myeloma*).ti,ab,kf. |
| 3 | (plasma adj2 cell adj2 myeloma*).ti,ab,kf. |
| 4 | (kahler adj2 disease*).ti,ab,kf. |
| 5 | (plasma-cell adj2 myeloma*).ti,ab,kf. |
| 6 | ("myeloma-multiple" or "myeloma-multiples").ti,ab,kf. |
| 7 | (myelomatoses or myelomatosis).ti,ab,kf. |
| 8 | leukemia, plasma cell/ |
| 9 | (plasma adj2 cell adj2 (leukemi* or leukaemi*)).ti,ab,kf. |
| 10 | (plasmacytic adj2 (leukemi* or leukaemi*)).ti,ab,kf. |
| 11 | or/1-10 |
| 12 | Frail Elderly/ |
| 13 | Frailty/ |
| 14 | (frail or frailer or frailty or vulnerabl*).ti,ab,kf. |
| 15 | (fragility or infirmity or infirmities or debility or debilities or incapacity or incapacities or delicacy or delicacies or indisposition* or decrepitude or decrepit).ti,ab,kf. |
| 16 | ((intermediat* or moderat*) adj2 fit).ti,ab,kf. |
| 17 | ("intermediate-fit " or "moderately-fit").ti,ab,kf. |
| 18 | ("non-fit" or "unfit").ti,ab,kf. |
| 19 | geriatrics/ |
| 20 | geriatrician*.ti,ab,kf. |
| 21 | geriatric assessment/ |
| 22 | (geriatric* adj2 (evaluat* or assess* or consult*)).ti,ab,kf. |
| 23 | (functional adj2 status adj2 (evaluat* or assess* or state or condition* or consult*)).ti,ab,kf. |
| 24 | or/12-23 |
| 25 | 11 and 24 |

*EMBASE (OvidSP)*

| **#** | **Searches** |
| --- | --- |
| 1 | multiple myeloma/ |
| 2 | (multiple adj2 myeloma*).ti,ab,kf. |
| 3 | (plasma adj2 cell adj2 myeloma*).ti,ab,kf. |
| 4 | (kahler adj2 disease*).ti,ab,kf. |
| 5 | (plasma-cell adj2 myeloma*).ti,ab,kf. |
| 6 | ("myeloma-multiple" or "myeloma-multiples").ti,ab,kf. |
| 7 | (myelomatoses or myelomatosis).ti,ab,kf. |
| 8 | plasma cell leukemia/ |
| 9 | (plasma adj2 cell adj2 (leukemi* or leukaemi*)).ti,ab,kf. |
| 10 | (plasmacytic adj2 (leukemi* or leukaemi*)).ti,ab,kf. |
| 11 | or/1-10 |
| 12 | Frail Elderly/ |
| 13 | Frailty/ |
| 14 | (frail or frailer or frailty or vulnerabl*).ti,ab,kf. |
| 15 | (fragility or infirmity or infirmities or debility or debilities or incapacity or incapacities or delicacy or delicacies or indisposition* or decrepitude or decrepit).ti,ab,kf. |
| 16 | ((intermediat* or moderat*) adj2 fit).ti,ab,kf. |
| 17 | ("intermediate-fit " or "moderately-fit").ti,ab,kf. |
| 18 | ("non-fit" or "unfit").ti,ab,kf. |
| 19 | geriatrics/ |
| 20 | geriatrician/ |
| 21 | geriatrician*.ti,ab,kf. |
| 22 | geriatric assessment/ |
| 23 | (geriatric* adj2 (evaluat* or assess* or consult*)).ti,ab,kf. |
| 24 | functional assessment/ |
| 25 | (functional adj2 status adj2 (evaluat* or assess* or state or condition* or consult*)).ti,ab,kf. |
| 26 | functional status assessment/ or clinical frailty scale/ |
| 27 | or/12-26 |
| 28 | 11 and 27 |

*Cochrane (Wiley)*

| **#** | **Searches** |
| --- | --- |
| 1 | multiple myeloma/ |
| 2 | (multiple adj2 myeloma*).ti,ab,kw. |
| 3 | (plasma adj2 cell adj2 myeloma*).ti,ab,kw. |
| 4 | (kahler adj2 disease*).ti,ab,kw. |
| 5 | (plasma-cell adj2 myeloma*).ti,ab,kw. |
| 6 | ("myeloma-multiple" or "myeloma-multiples").ti,ab,kw. |
| 7 | (myelomatoses or myelomatosis).ti,ab,kw. |
| 8 | plasma cell leukemia/ |
| 9 | (plasma adj2 cell adj2 (leukemi* or leukaemi*)).ti,ab,kw. |
| 10 | (plasmacytic adj2 (leukemi* or leukaemi*)).ti,ab,kw. |
| 11 | or/1-10 |
| 12 | Frail Elderly/ |
| 13 | Frailty/ |
| 14 | (frail or frailer or frailty or vulnerabl*).ti,ab,kw. |
| 15 | (fragility or infirmity or infirmities or debility or debilities or incapacity or incapacities or delicacy or delicacies or indisposition* or decrepitude or decrepit).ti,ab,kw. |
| 16 | ((intermediat* or moderat*) adj2 fit).ti,ab,kw. |
| 17 | ("intermediate-fit " or "moderately-fit").ti,ab,kw. |
| 18 | ("non-fit" or "unfit").ti,ab,kw. |
| 19 | geriatrics/ |
| 20 | geriatrician*.ti,ab,kw. |
| 21 | geriatric assessment/ |
| 22 | (geriatric* adj2 (evaluat* or assess* or consult*)).ti,ab,kw. |
| 23 | functional assessment/ |
| 24 | (functional adj2 status adj2 (evaluat* or assess* or state or condition* or consult*)).ti,ab,kw. |
| 25 | or/12-24 |
| 26 | 11 and 25 |

*Web of Science (Clarivate)*

| **#** | **Searches** |
| --- | --- |
| 1 | TS=(multiple NEAR/2 myeloma*)OR TS=(plasma NEAR/2 cell NEAR/2 myeloma*) OR TS=(kahler NEAR/2 disease*) OR TS=("myeloma-multiple" OR "myeloma-multiples" OR myelomatoses OR myelomatosis) |
| 2 | TS=(plasma NEAR/2 cell NEAR/2 (leukemi* or leukaemi*)) OR TS=(plasmacytic NEAR/2 (leukemi* OR leukaemi*)) |
| 3 | #2 OR #1 |
| 4 | TI=(frail OR frailer OR frailty OR vulnerabl* OR fragility OR infirmity OR infirmities OR debility OR debilities OR incapacity OR incapacities OR delicacy OR delicacies OR indisposition* OR decrepitude OR decrepit) |
| 5 | TI=((intermediat* or moderat*) NEAR/2 fit) OR TI=("intermediate-fit " OR "moderately-fit") OR TI=("non-fit" or "unfit") |
| 6 | AB=(frail OR frailer OR frailty OR vulnerabl* OR fragility OR infirmity OR infirmities OR debility OR debilities OR incapacity OR incapacities OR delicacy OR delicacies OR indisposition* OR decrepitude OR decrepit) |
| 7 | AB=((intermediat* or moderat*) NEAR/2 fit) OR AB=("intermediate-fit " OR "moderately-fit") OR AB=("non-fit" or "unfit") |
| 8 | TI=(geriatric* NEAR/2 (evaluat* or assess* or consult*)) |
| 9 | AB=(geriatric* NEAR/2 (evaluat* or assess* or consult*)) |
| 10 | TI=(functional NEAR/2 status NEAR/2 (evaluat* or assess* or state or condition* or consult*)) |
| 11 | AB=(functional NEAR/2 status NEAR/2 (evaluat* or assess* or state or condition* or consult*)) |
| 12 | TI=(geriatric or geriatrics or geriatrician*) |
| 13 | AB=(geriatric or geriatrics or geriatrician*) |
| 14 | #4 OR #5 OR #6 OR #7 OR #8 OR #9 OR #10 OR #11 OR #12 OR #13 |
| 15 | #14 AND #3 |

[*Scopus*](https://www-webofscience-com.libaccess.lib.mcmaster.ca/wos/woscc/summary/93a7a712-0064-4f51-876e-5a52a8b827bb-2ed3bdfe/relevance/1) *(Elsevier)*

| **#** | **Searches** |
| --- | --- |
| 1 | ( TITLE-ABS-KEY ( frail  OR  frailer  OR  frailty  OR  vulnerabl*  OR  fragility  OR  infirmity  OR  infirmities  OR  debility  OR  debilities  OR  incapacity  OR  incapacities  OR  delicacy  OR  delicacies  OR  indisposition*  OR  decrepitude  OR  decrepit  OR  "intermediate-fit"  OR  "moderately-fit"  OR  "non-fit"  OR  unfit )  AND  TITLE-ABS-KEY ( multiple  AND myeloma* ) ) |
| 2 | ( TITLE-ABS-KEY ( frail  OR  frailer  OR  frailty  OR  vulnerabl*  OR  fragility  OR  infirmity  OR  infirmities  OR  debility  OR  debilities  OR  incapacity  OR  incapacities  OR  delicacy  OR  delicacies  OR  indisposition*  OR  decrepitude  OR  decrepit  OR  "intermediate-fit"  OR  "moderately-fit"  OR  "non-fit"  OR  unfit )  AND  TITLE-ABS-KEY ( ( plasma  W/2  cell  W/2  myeloma* ) ) ) |
| 3 | ( TITLE-ABS-KEY ( ( geriatric* W/2 ( evaluat* OR assess* OR consult* ) ) OR ( ( intermediat* OR moderat* ) W/2 fit ) ) AND TITLE-ABS-KEY ( ( plasma W/2 cell W/2 myeloma* ) ) ) |
| 4 | ( TITLE-ABS-KEY ( ( geriatric*  W/2  ( evaluat*  OR  assess*  OR  consult* ) )  OR  ( ( intermediat*  OR  moderat* )  W/2  fit ) )  AND  TITLE-ABS-KEY ( multiple  AND myeloma* ) ) |
| 5 | 1 or 2 or 3 or 4 |
